# Polyether Ionophore Antibiotics Target Drug-Resistant Clinical Isolates, Persister Cells, and Biofilms

**DOI:** 10.1128/spectrum.00625-23

**Published:** 2023-06-08

**Authors:** Malene Wollesen, Kasper Mikkelsen, Marie Selch Tvilum, Martin Vestergaard, Mikala Wang, Rikke L. Meyer, Hanne Ingmer, Thomas B. Poulsen, Thomas Tørring

**Affiliations:** a Department of Chemistry, Aarhus University, Aarhus, Denmark; b Department of Biological and Chemical Engineering, Aarhus University, Aarhus, Denmark; c Department of Veterinary and Animal Sciences, University of Copenhagen, Frederiksberg, Denmark; d Department of Clinical Microbiology, Aarhus University Hospital, Palle Juul-Jensens, Aarhus, Denmark; e Interdisciplinary Nanoscience Center, Aarhus University, Aarhus, Denmark; f Department of Biology, Aarhus University, Aarhus, Denmark; Ludwig-Maximilians-Universitat Munchen Pettenkofer Institute

**Keywords:** ionophore, antibiotics, biofilm, persister cells, antimicrobial resistance, *Staphylococcus aureus*, natural antimicrobial products

## Abstract

Polyether ionophores are complex natural products known to transport various cations across biological membranes. While several members of this family are used in agriculture (e.g., as anti-coccidiostats) and have potent antibacterial activity, they are not currently being pursued as antibiotics for human use. Polyether ionophores are typically grouped as having similar functions, despite the fact that they significantly differ in structure; for this reason, how their structure and activity are related remains unclear. To determine whether certain members of the family constitute particularly interesting springboards for in-depth investigations and future synthetic optimization, we conducted a systematic comparative study of eight different polyether ionophores for their potential as antibiotics. This includes clinical isolates from bloodstream infections and studies of the compounds’ effects on bacterial biofilms and persister cells. We uncover distinct differences within the compound class and identify the compounds lasalocid, calcimycin, and nanchangmycin as having particularly interesting activity profiles for further development.

**IMPORTANCE** Polyether ionophores are complex natural products used in agriculture as anti-coccidiostats in poultry and as growth promoters in cattle, although their precise mechanism is not understood. They are widely regarded as antimicrobials against Gram-positive bacteria and protozoa, but fear of toxicity has so far prevented their use in humans. We show that ionophores generally have very different effects on Staphylococcus aureus, both in standard assays and in more complex systems such as bacterial biofilms and persister cell populations. This will allow us to focus on the most interesting compounds for future in-depth investigations and synthetic optimizations.

## INTRODUCTION

The number of infections caused by antimicrobial-resistant microorganisms is rising and poses a major threat to society. One widely discussed survey from antimicrobial resistance collaborators, published in early 2022, estimated that 4.97 million deaths globally were associated with antimicrobial resistance (AMR) in 2019 alone (1). The majority of currently used antibiotics target a limited number of processes common to most microorganisms: cell wall integrity (beta-lactams and glycopeptides), cell membrane (lipopeptides), translation (chloramphenicol and macrolides), transcription (rifamycins), and DNA replication (fluoroquinolones). Several papers have argued that new antimicrobial treatments may be discovered if we can alter the bacterial metabolic state, as many antibiotics are only bactericidal against metabolically active bacteria (2–4). This is exemplified by the increase in bacterial aminoglycoside uptake stimulated by the addition of simple metabolites, such as fructose, mannitol, or glucose, the catabolism of which increases the proton motive force (PMF) and electric potential across the membrane (ΔΨ) and kills otherwise dormant bacteria (5). Similarly, it has been reported that differences in bicarbonate concentration between an infection and typical *in vitro* screening in Mueller-Hinton broth can affect drug translatability. Farha et al. elegantly showed that this is probably because bicarbonate dissipates the ΔpH component of the PMF, causing a compensatory increase in the ΔΨ (6, 7).

These deviations from a simplistic model of one antibiotic blocking a single essential process beg a more nuanced view of bacterial physiology during infections and the ultimate causes of bacterial death beyond the target-focused proximal causes, as discussed by Baquero and Levin (8).

Polyether ionophores, natural products typically produced by *Streptomycetes*, fit this particular description well. They are a large class of structurally diverse amphiphilic antibiotics with the ability to perturb cellular ion gradients by affecting the transmembrane electroneutral exchange of ions/protons down a concentration gradient (9–11). The molecules achieve this by undergoing a dynamic conformational change upon binding to the cation. This initial ion complex then undergoes a conformational change to fully encapsulate the bound ion by orienting multiple polar groups toward the cavity interior. Conversely, hydrophobic groups are directed to the exterior, allowing diffusion of the ionophore-ion complex across the lipid bilayer (12). The action of ionophores in (bacterial) cells depends on multiple parameters that transcend this simple, canonical mechanism. Depending on their specific structure, ionophores have differential affinities for different cations and likely have differing ion-transport dynamics and membrane affinities. As such, exposure to a given ionophore constitutes a unique perturbation of both metal-ion and proton gradients that a bacterium would attempt to counteract. Polyether ionophores can also affect eukaryotic cells, and while some compounds are used extensively as oral antiparasitic agents in agriculture, the development of ionophore antibiotics for human use has not been pursued due to the risk of toxicity. This is clearly a multifaceted situation; however, the best interpretation currently is that many organisms can tolerate polyether ionophores and that dosing schedules can be adapted to increase compliance (13). Despite their extensive use in animals, there is no evidence of cross-resistance between polyether ionophores and other antibiotics, which is interesting in relation to antimicrobial resistance (14).

We hypothesize that the differences in membrane composition between mammalian and bacterial cells can be exploited to prepare ionophores with acceptable therapeutic indices for use in certain clinical scenarios to treat resistant bacterial infections in humans. Recently, as an initial milestone toward this end, we described synthetic polyether ionophores with enhanced inhibitory selectivity for bacterial cells versus mammalian cells compared to a series of benchmarked natural products (15). In the present study, we systematically compared eight members of the natural polyether ionophore class (Fig. S1 in the supplemental material). While many of these agents have been subject to earlier investigation for their antibiotic properties, these data are often generated under different experimental conditions, which we suspected might confound similarities (or differences) between the compounds. Furthermore, we demonstrate for the first time the antimicrobial properties of this class of compounds against bacterial biofilms and persister cells. Our data demonstrate surprising differences between members of this family of antibiotics and, furthermore, uncover inhibitory profiles for selected compounds that prompt investigations in preclinical infection models and further structure activity relationship (SAR) investigations.

## RESULTS AND DISCUSSION

### Polyether ionophores are effective across a panel of *Staphylococcus aureus* strains from bloodstream infections.

**FIG 1:**
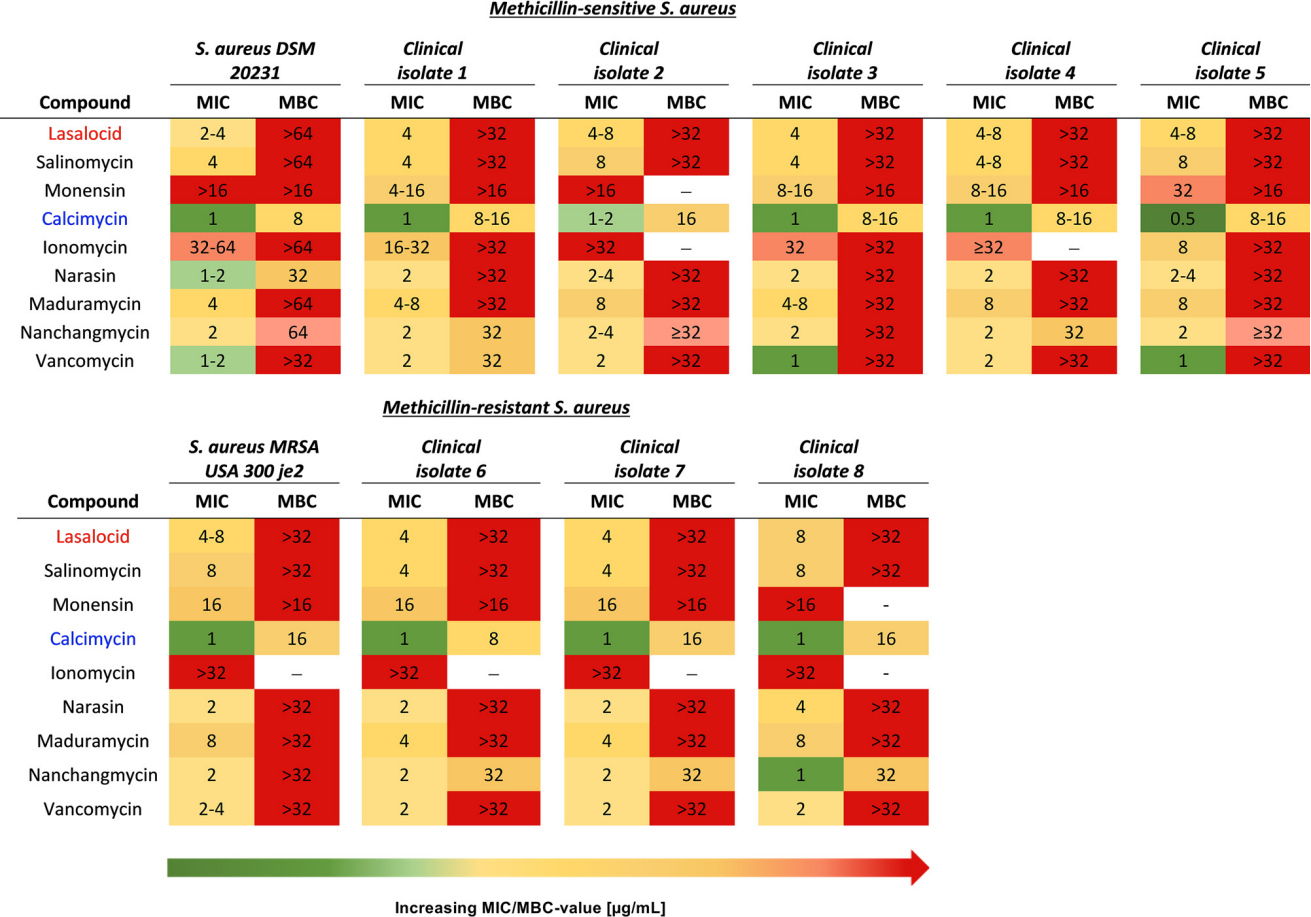
Polyether ionophore bioactivity in methicillin-sensitive (MSSA) and methicillin-resistant S. aureus (MRSA) strains. Polyether S. aureus isolates from bloodstream infections were obtained from Aarhus University Hospital (AUH), Denmark. Five methicillin-sensitive S. aureus strains (A) and three MRSA strains (B) were obtained, and bioactivity was determined using MIC and MBC analyses in a defined minimal medium M9 (CM-M9). All results are listed as the obtained concentration ranges (μg/mL) of at least two biological repetitions, each with technical triplicates (distinct wells).

To immediately establish clinical relevance for investigating ionophores as antibacterial compounds against Staphylococcus aureus, we determined the minimum inhibitory concentration (MIC) and the minimum bactericidal concentration (MBC) for several natural product ionophores against both methicillin-sensitive (MSSA) and methicillin-resistant (MRSA) S. aureus clinical isolates (Fig. 1, Table S4). Two observations stand out from these experiments: (i) all ionophores were equally effective across all tested strains, and (ii) all ionophores had a large difference between relatively low MIC values and MBC values that were not measurable in the tested range, making the compounds bacteriostatic. A similar activity profile was observed with the vancomycin control. The only exception was calcimycin, a structurally atypical polyether ionophore (16).

**FIG 2:**
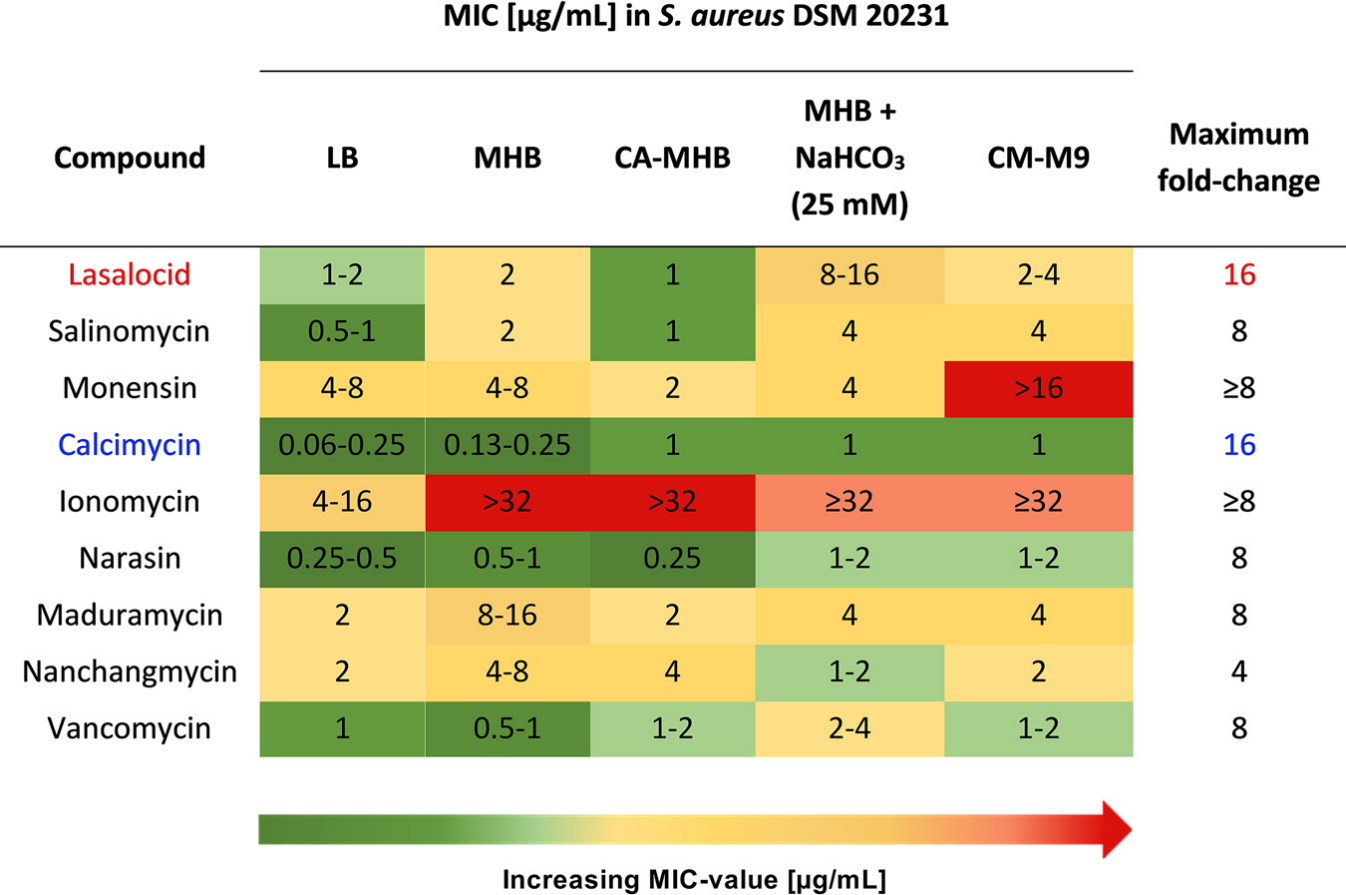
MIC of polyether ionophores in S. aureus in the presence of different growth media. MIC determination was performed in technical triplicates and biological replicates in 96-well plates in accordance with CLSI recommendations.

We conducted these experiments in a defined minimal medium CM-M9 (M9 salts with Ca^2+^ and Mg^2+^ adjusted to physiological levels) and, serendipitously, noticed a large discrepancy between these values and those previously reported using type strains in Lysogeny broth (LB) (15). Due to the rather broad mechanistic role of polyether antibiotics as ion transporters, we suspected that the medium composition might play a significant role. Consequently, we determined the MIC values of our polyether ionophore panel in a small set of commonly used media: Lysogeny broth, Mueller-Hinton broth (MHB), cation-adjusted MHB (CA-MHB), and the defined minimal medium M9 (CM-M9) (see Fig. 2). Compared to vancomycin, whose activity varied from 0.5 to 4 μg/mL depending on the medium composition (maximum 8-fold change), some of the ionophores appeared more sensitive to change (more than 8-fold). Apart from maduramycin and nanchangmycin, the ionophores had the lowest MIC value in LB and the highest in CM-M9. A similar trend has been reported for calcimycin and ionomycin against Bacillus subtilis (17). We also determined the MIC in MHB supplemented with sodium bicarbonate (25 mM) to approach more clinically relevant screening conditions because supplementation with sodium bicarbonate has been shown to improve the *in vitro* to *in vivo* translatability of MHB (6). The MIC values mirrored those determined for the CM-M9 medium. Because the chemically defined CM-M9 allows much better control over concentration of individual cations we chose to proceed with this medium in the subsequent experiments.

### The antibiotic properties of ionophores are affected by cation concentration.

**FIG 3:**
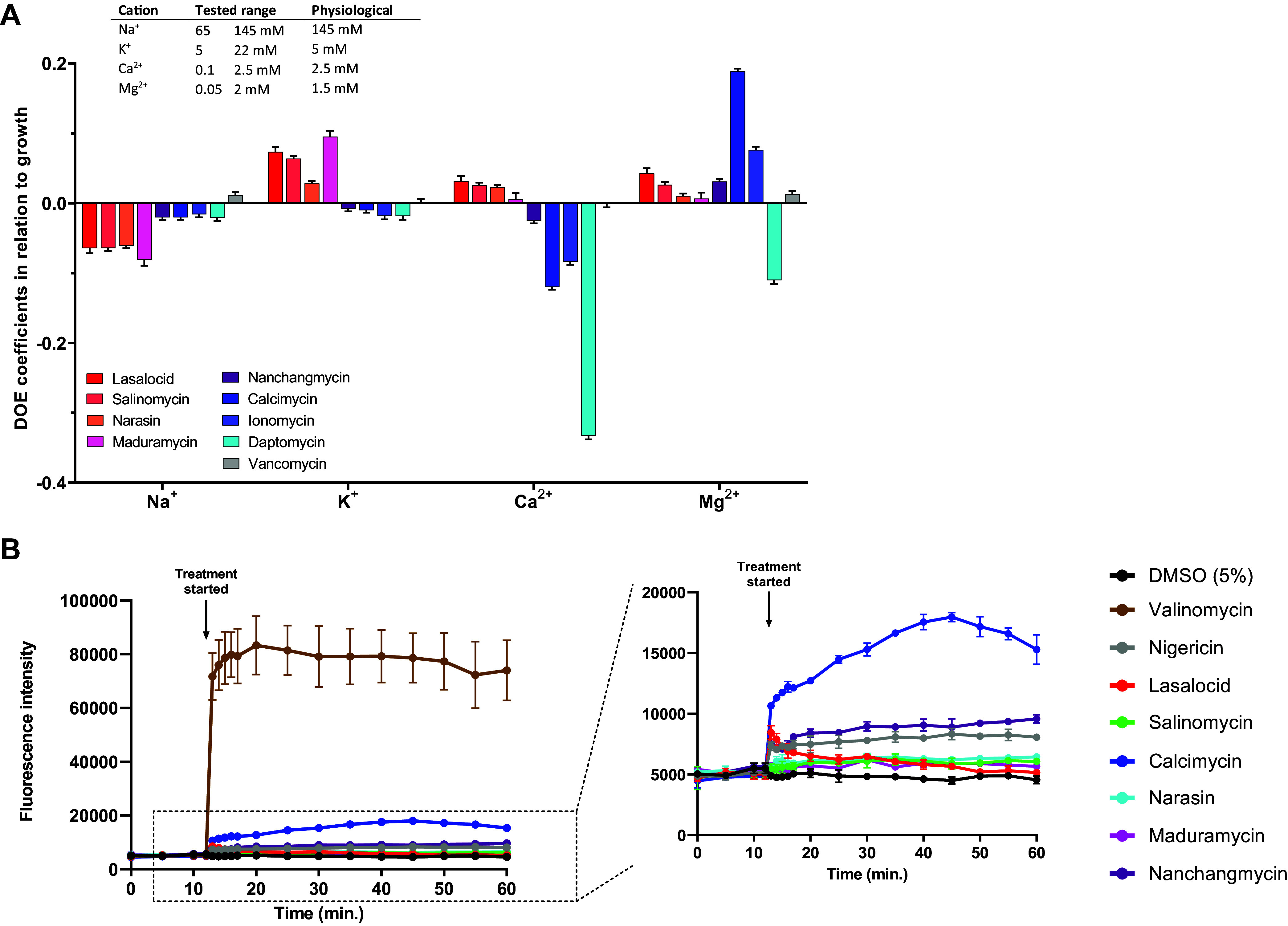
Cation dependency of polyether ionophores. (A) A full factorial design of experiment (DOE)-approach was used to study how ionophore activity depends on cation concentrations (Na^+^, K^+^, Ca^2+^, and Mg^2+^). The concentrations of the four cations were chosen as factors, and the percentage change in growth compared to a control as the response. S. aureus was treated with the compounds at 0.25× MIC (1× MIC for daptomycin) in the presence of cations in the concentration ranges listed in the figure. Bacterial growth was evaluated after overnight treatment, and MODDE Pro was used for data processing. Daptomycin and vancomycin were included as positive and negative controls, respectively. Results are average of technical triplicates. (B) Ionophore effects on membrane potential were studied using the membrane-potential sensitive dye 3,3-dipropylthiacarbocyanine iodide [DiSC_3_(5)]. S. aureus was loaded with 1 μM DiSC_3_(5) (1%), and after the dye had stabilized, the culture was treated with 10× MIC (CM-M9 medium). Valinomycin (20 μg/mL) and nigericin (10 μg/mL) were included as positive and negative controls, respectively. Results are average of technical triplicates (distinct wells), and bars are means ± SD (standard deviation) (*n* = 3).

Based on the ionophores’ fluctuation in inhibitory potency observed between different media and their general role in ion transport, we hypothesized that cation concentrations would modulate their activity. This prompted us to systematically investigate the effect of changing the concentrations of Na^+^, K^+^, Mg^2+^, and Ca^2+^ in a full factorial design. Experimentally, the cation concentrations were set to vary within physiologically relevant ranges in a CM-M9-based medium. The ranges were set as follows: Na^+^, 65 to 125 mM; K^+^, 5 to 25 mM; Mg^2+^, 0.05 to 2 mM; and Ca^2+^, 0.1 to 2.5 mM; and we used growth as the response variable upon challenge with ionophores at 0.25×, 1×, or 4× MIC (Fig. 3A). To validate the experimental setup, we also tested daptomycin, which should exhibit calcium dependence, and vancomycin, which we did not expect to be influenced by changes in cation concentration within the set ranges. Indeed, daptomycin was potentiated with increasing calcium concentration and, to a lesser extent, increasing magnesium concentration. Vancomycin inhibition showed a slight inverse correlation with sodium concentration, but generally negligible cation dependency, as expected (Fig. 3A). Compared to that of daptomycin, the ionophores’ inhibitory effects were generally less impacted by changing cation concentrations and fell into two broad groups: calcimycin and ionomycin inhibition correlated with *increasing* calcium concentrations and, interestingly, with *decreasing* magnesium concentrations, whereas lasalocid, salinomycin, narasin, and maduramycin inhibition correlated with increasing sodium and decreasing potassium concentrations. Calcimycin has also been reported to drastically lower the tolerated amount of added iron, manganese, and calcium to B. subtilis (17). These data largely reflect the affinity toward either monovalent (salinomycin, monensin, narasin) or divalent cations (calcimycin, ionomycin) that have been historically reported (18), although it is also clear that each compound has a unique profile.

As a consequence of their dual ion-transport and membrane-binding activities, polyether ionophores may acutely disrupt cellular membrane potential. This effect could be the cause of both their anti-bacterial and cytotoxic effects. To probe the effect of ionophores on membrane potential, we used the dye 3,3-dipropylthiacarbocyanine iodide [DiSC_3_(5)]. DiSC_3_(5) aggregates within the bacterial membrane, causing self-quenching of its fluorescence; however, upon disruption of the membrane electrical potential (ΔΨ), the dye is released into the medium, accompanied by an increase in fluorescence intensity. We tested eight naturally occurring polyether ionophores at 10× MIC in this setup (Fig. 3B). As a positive control, we used the pore-forming K^+^ ionophore valinomycin, which is known to dissipate the ΔΨ. Compared to valinomycin, the polyether ionophores did not dissipate ΔΨ, as the fluorescence intensity remained stable for 60 min (Fig. 3B). Only calcimycin caused a slight increase in fluorescence intensity, although much less than valinomycin. Work by Farha et al. (19) has shown that DiSC_3_(5) can indirectly probe the proton gradient (ΔpH) across the membrane because bacteria respond to a decreasing ΔpH by increasing ΔΨ, thereby leading to decreased fluorescence readout by DiSc_3_(5). The latter effect was reported previously for nigericin, but we did not observe any significant response for the ionophores tested here. In conclusion, our data indicate that the ionophores do not disrupt the membrane potential in any significant way. Another study by Farha et al.(7) showed that the drastic changes in antibiotic potency at increased sodium bicarbonate levels are caused by a selective dissipation of the ΔpH. They also showed that dissipators of ΔΨ (valinomycin, I1, I2, and I3) act synergistically with bicarbonate. If the ionophores did in fact function as potent dissipators of membrane potential, we should see a more pronounced effect on the measured MIC values when adding bicarbonate.

### Selected polyether ionophores kill bacterial persisters and biofilms.

**FIG 4:**
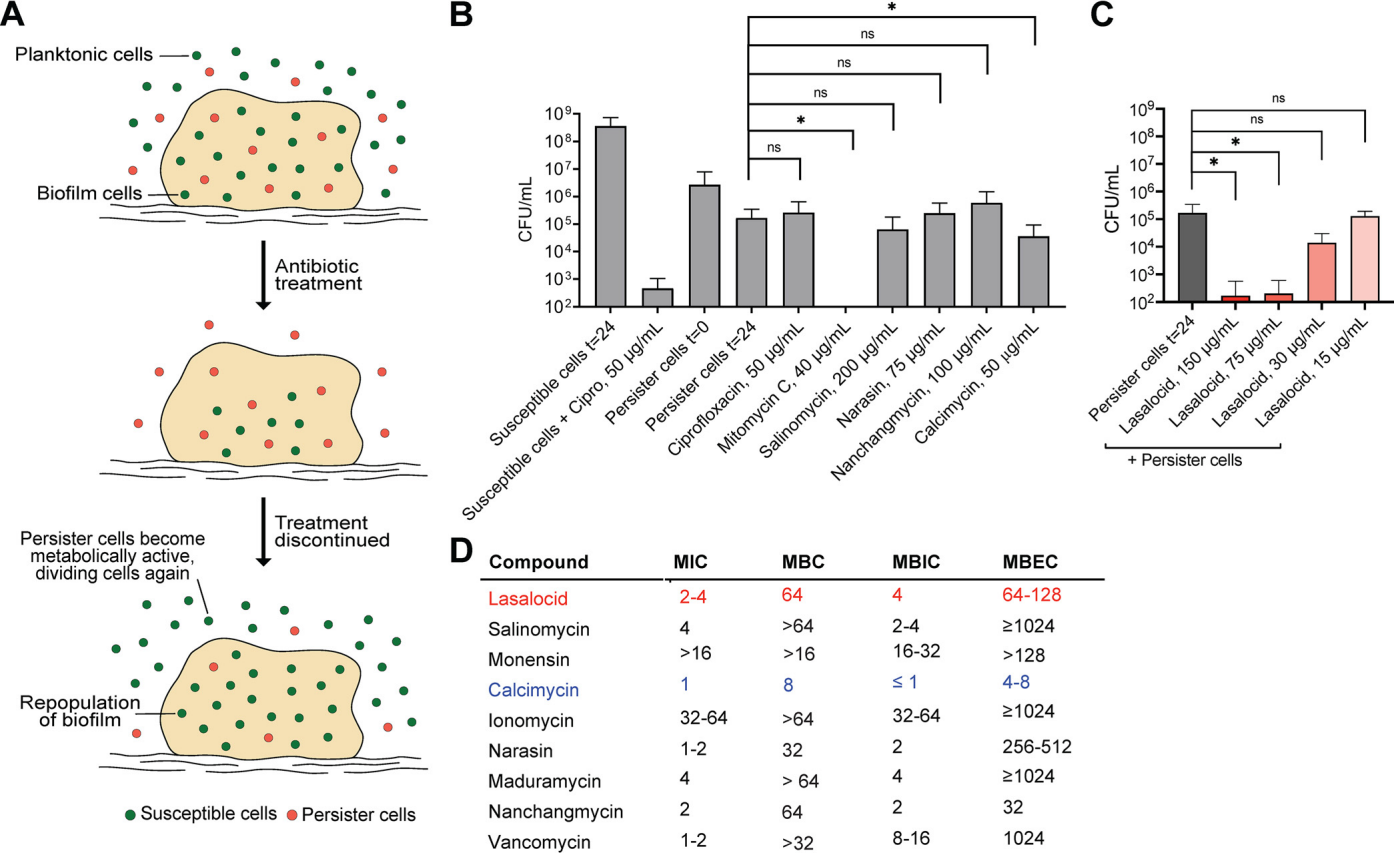
Polyether ionophore action on S. aureus persister cells and biofilms. (A) Illustration showing the lack of antibiotic effect against persister and biofilm cells. (B) Persister cell killing with 50× MIC (the respective concentrations are listed in the figure). Persister cells were produced by transferring growing, susceptible bacteria into a modified CM-M9 medium without available carbon sources. Persister cells reverted to susceptible cells when transferred into a nutrient-rich medium (susceptible cells *t* = 24). Ciprofloxacin (50 μg/mL) and mitomycin C (40 μg/mL) were included as negative and positive controls, respectively. Bars are mean ± SD (*n* = 9). (C) Persister cell killing with lasalocid at 5×, 10×, 25×, and 50× MIC. Bars are means ± SD (*n* = 9). (D) Biofilm inhibition and eradication. A S. aureus biofilm was grown for 24 h before antibiotic treatment. Minimum biofilm inhibition concentration (MBIC) was determined after 24 h of treatment, and minimum biofilm eradication concentration (MBEC) was determined after 72 h of biofilm recovery. Lasalocid (red) and calcimycin (blue) are highlighted for clarity. All results shown are averages of biological triplicates, each with technical triplicates.

We next investigated antibacterial effects beyond simple early exponential growth. Two of the most substantial clinical challenges related to infections are the formation of biofilms and persister cells, both of which are primary causes of chronic and recurrent infections (20–23). Upon antibiotic treatment, bacterial cells can transform into metabolically inactive persister cells, making them highly resilient to treatment and allowing them to “escape” the antibiotic (Fig. 4A). When antibiotic treatment is discontinued, the persister cells eventually transition back into metabolically active cells, creating recurrent infections. Biofilms, which typically grow on surfaces of medical implants or host tissue, are characterized by increased tolerance to antibiotic treatment. This increased tolerance is attributed to a lower growth rate, decreased antibiotic diffusion, higher oxidative stress tolerance, and a larger subpopulation of persister cells (Fig. 4A) (21). To investigate the effects of polyether ionophores on metabolically inactive persister cells, we used a recently developed high-throughput method which screens the ability to kill non-growing cells by performing the test without a carbon source (24). In short, we transferred S. aureus cells to CM-M9 medium with the carbon source omitted—preventing any growth and mimicking the subpopulation left after antibiotic treatment—and incubated them with antibiotics for 24 h before determining the viable cell count. We used ciprofloxacin (50 μg/mL) as the negative control (no inhibitory activity against persister cells) and the DNA-cross-linker mitomycin C (40 μg/mL) as the positive control for killing persister cells. When tested at 50× MIC, the ionophores displayed very different inhibitory potential in this context (Fig. 4B). While most compounds were completely inactive, calcimycin showed a modest effect on the persister cells at 50× MIC, but lasalocid showed a concentration-dependent inhibitory effect on the persister cells and a remarkable potency at >75 μg/mL. Next, using a 96-well assay based on peg-lids, we determined the minimum biofilm inhibition concentration (MBIC) and minimum biofilm eradication concentration (MBEC) for the ionophores (Fig. 4D). For most of the compounds, the MBIC mirrored the MIC. The MBECs of most of the compounds were very high, which is in agreement with a previous report on S. aureus on some of these ionophores (25). However, lasalocid, nanchangmycin, and calcimycin stood out with promising MBEC values similar to their MBC, indicating that under these test conditions, the biofilm matrix and the persister phenotype in these biofilms do not sufficiently protect the bacteria against the antimicrobial action of the ionophores; however, the proximate cause of this is still unclear.

### Time-dependent killing of *S. aureus*.

**FIG 5:**
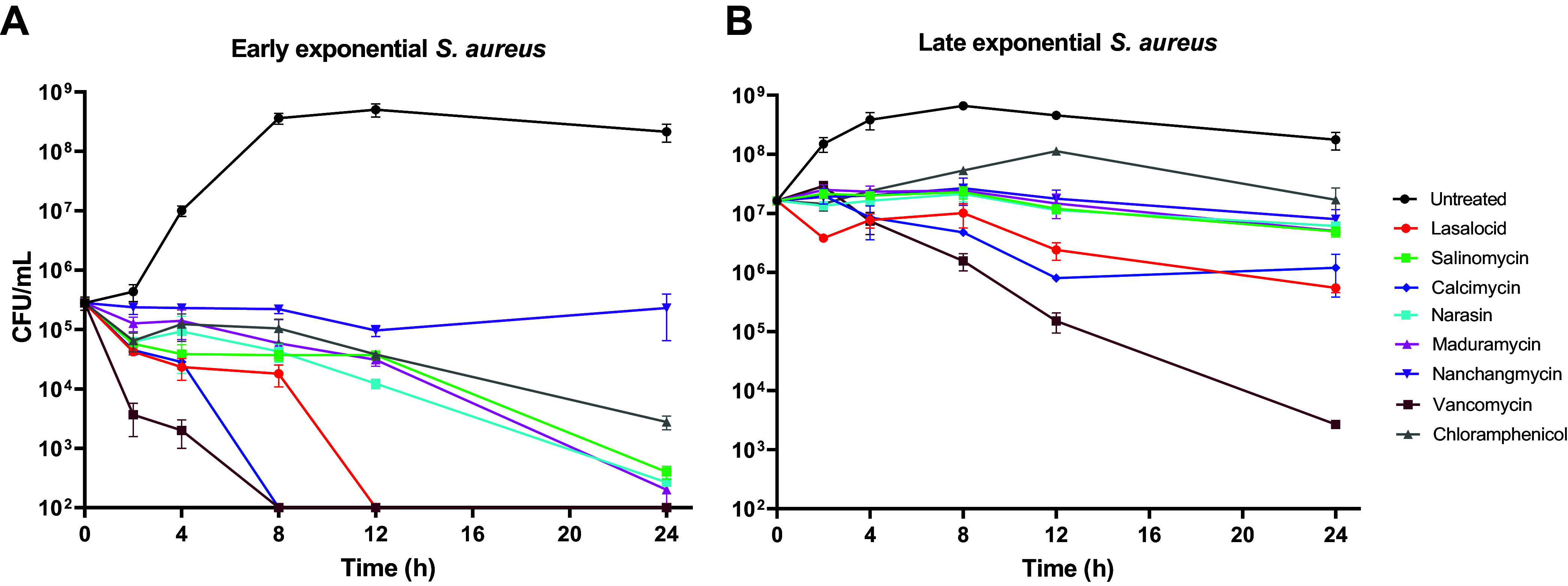
Time-dependent killing of S. aureus. S. aureus was grown to early (A) and late (B) exponential phase and treated with ionophores at 10× MIC (in CM-M9 medium) for 0, 2, 4, 8, 12, and 24 h, at which point cells were harvested and CFU/mL was enumerated (cutoff: 10^2^ CFU/mL). Vancomycin (10× MIC of 2 μg/mL) and chloramphenicol (1× MIC of 16 μg/mL) were included as bactericidal and bacteriostatic controls, respectively. Individual data points are means ± SD (*n* = 3) of technical triplicates (distinct wells). Figure is representative of two biological replicates.

After establishing the dependencies on medium composition and cation concentration, we challenged S. aureus with the ionophores (10× MIC) in a time-dependent fashion (Fig. 5) and compared their activity to that of chloramphenicol and vancomycin. In an early exponential-phase culture (5 × 10^5^ CFU/mL), all ionophores except for nanchangmycin displayed bactericidal behavior. Lasalocid and calcimycin caused a rapid decline in CFU that dropped below our level of detection within just 12 h. The same two compounds also showed the greatest effect in a late exponential-phase culture (2 × 10^7^ CFU/mL). This also reflects the rather large difference in MIC and MBC that we observed for many of the ionophores. In conclusion, when the effects in the biofilm and persister assays and time-dependent killing are viewed in combination, nanchangmycin, lasalocid, and calcimycin stand out as promising for further investigation as antibiotic leads.

### Genes affecting ionophore susceptibility.

**FIG 6:**
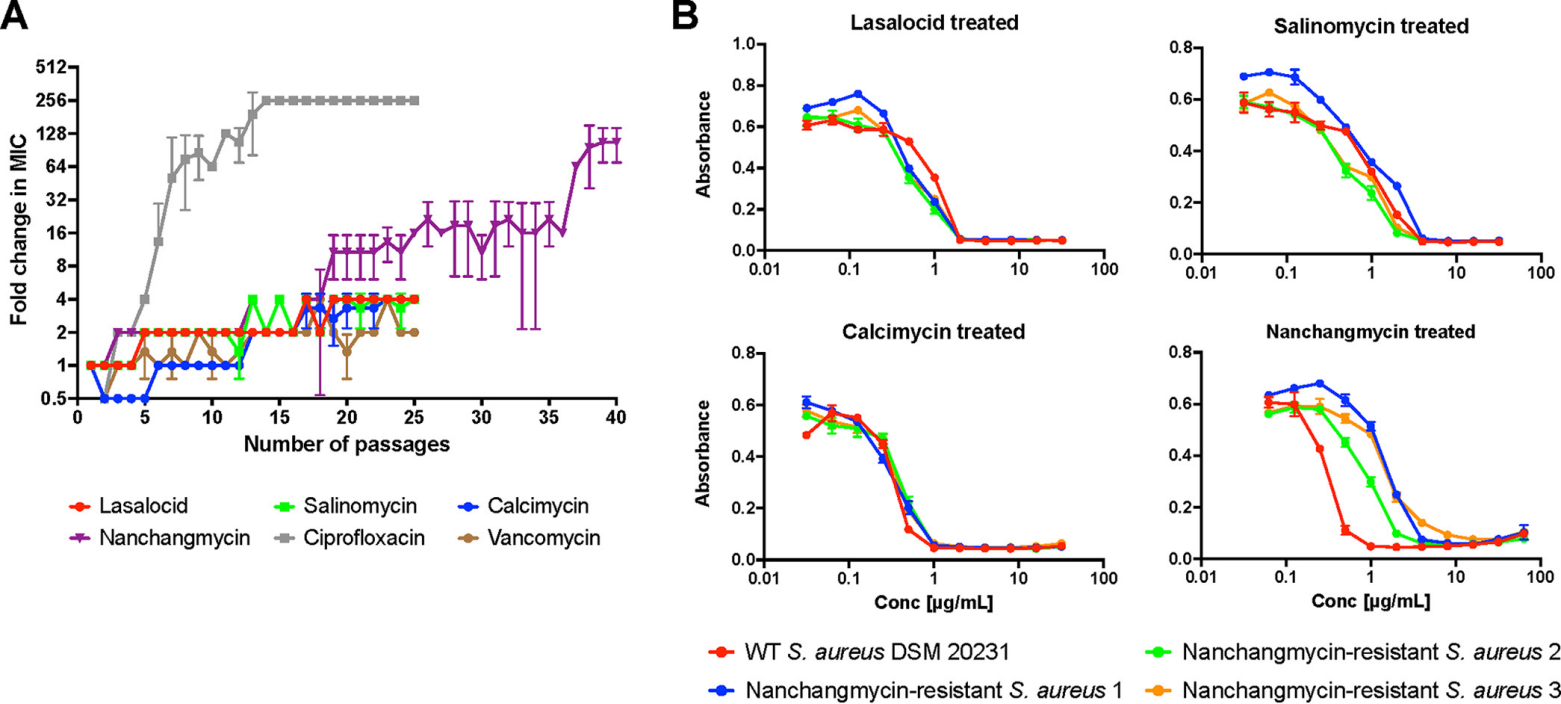
Resistance studies. (A) Resistance toward ionophores was developed with a sequential passaging method. S. aureus was treated with five ionophore concentrations (0.25× to 4× MIC), and the MIC was determined each day. The culture grown at the highest concentration (0.5× new MIC) was passed on to the following day and treated with concentrations relative to the new MIC value. This was continued for 25 to 40 days. Ciprofloxacin and vancomycin were included as positive and negative controls, respectively. Data points are means ± SD (*n* = 3). (B) The three nanchangmycin-treated cultures on day 40 in panel A were subjected to MIC analysis to confirm resistance and checked for cross-resistance toward the other three ionophores. Wild-type S. aureus DSM 20231 was included as a control strain. Data points are means ± SD (*n* = 3), and results are a representative of biological duplicates.

When assessing the potential of polyether ionophores as antibiotic leads, one important aspect to address is the frequency of resistance, especially considering that many ionophores are used in agriculture. In addition, this can also provide insights into an antibiotic’s mechanism of action, as mutations which confer resistance can reveal the compound’s target. The organisms which produce the ionophores often contain genes conferring self-resistance. In the case of the ionophore tetronasin, Linton et al. (26) showed that two genes encoding a ATP-dependent efflux system in Streptomyces longisporoflavus conferred resistance to tetronasin when transferred to otherwise susceptible Streptomyces lividans and Streptomyces albus. Similarly, the biosynthetic gene clusters associated with monensin (BGC0000100), lasalocid (BGC0000087), and nanchangmycin (BGC0000105) found in the MIBiG (Minimum Information about a Biosynthetic Gene Cluster) database (27) all contain predicted efflux systems, indicating that this is the dominant resistance mechanism among the producing strains. To investigate this in a human pathogen, we examined resistance against selected ionophores: first, unsuccessfully, in the standard single-step challenge of a dense inoculum on agar plates containing 1× MBC, and next by a sequential passaging method in which an S. aureus culture was treated with sub-inhibitory concentrations every day for 4 weeks (Fig. 6). In this type of experiment, as the bacteria become less susceptible over time, and perhaps resistant, the MIC value will increase. However, after 25 days of continuous treatment, the S. aureus culture was barely affected by the exposure. For lasalocid, salinomycin, and calcimycin, only a 4-fold increase in the tolerated concentration was observed, which was comparable to the vancomycin control. In comparison, resistance to ciprofloxacin developed rapidly, as expected. These data mirror prior observations in the literature in which resistance toward polyether ionophores is not easily achieved and that their frequency of resistance is very low considering their extensive use (28). Interestingly, the S. aureus culture treated with nanchangmycin was slightly more affected after the first 25 days of treatment, with a 16-fold change in inhibitory concentration, and this treatment was therefore continued. After 40 days of continuous treatment, susceptibility was decreased, resulting in an apparent 64- to 128-fold increase in the MIC. However, when isolating single colonies to determine the final MIC values of the resistant strains, a modest 4- to 16-fold increase in the MIC was observed compared to the wild-type S. aureus strain (Fig. 6B). The nanchangmycin-resistant strains were checked for cross-resistance toward the other three ionophores, as this could have indicated whether a shared resistance mechanism was involved. However, none of the isolates showed any difference in tolerance to lasalocid, salinomycin, or calcimycin, thereby ruling out a common acquired resistance phenotype. We sequenced the genomes of three resistant strains and that of the wild-type S. aureus strain, which revealed several genes with mutations common to all three mutants and absent from the wild type (see Table S2). Among these were the gene encoding TrkH (WP_000021864.1), involved in potassium uptake; the gene encoding MspA (WP_001161085.1), a protein hypothesized to be membrane-stabilizing (29); and the gene encoding the transcriptional regulator SarV (WP_000066900.1), which is involved in the autolysis of S. aureus (30). Several mutations in both *mspA* and *sarV* were common to strains 1 and 3, while strain 2 only showed one mutation in each. These results matched previous reports that acquired ionophore-resistance is linked to efflux or decreased permeability (31, 32), but the proximate cause remains elusive. To gain additional insight into the ionophores’ mechanism of action, we screened the Nebraska Transposon Mutant Library (NTML), a mutant library in an MRSA strain with transposons inserted into 1,920 individual non-essential genes, for increased sensitivity to lasalocid, salinomycin, calcimycin, and nanchangmycin in an agar plate assay. Here, we primarily found hits with perturbations in the electron transport chain to be more sensitive to the ionophores (see Table S3). Measuring the MICs of the mutants in a liquid-based assay revealed that inactivation of *aroC* (encoding chorismate synthase) or *hemB* (encoding delta-aminolevulinic acid dehydratase) had the biggest effect on ionophore sensitivity, while mutations in genes of the quinol oxidase subunits I, II, or III (*qoxABC*), NADH:ubiquinone reductase (*ndh2*), or protoheme IX farnesyltransferase (*cyoE*) produced 2-fold decreases in the MICs of lasalocid, salinomycin, and nanchangmycin. None of the mutations influenced calcimycin sensitivity in a liquid setting. We have previously observed that many of these transposon mutants have a reduced membrane potential (33) and *hemB* has also been associated with small colony variants (34).

### Polyether ionophores synergize with polymyxin B to target Gram-negative pathogens.

**FIG 7:**
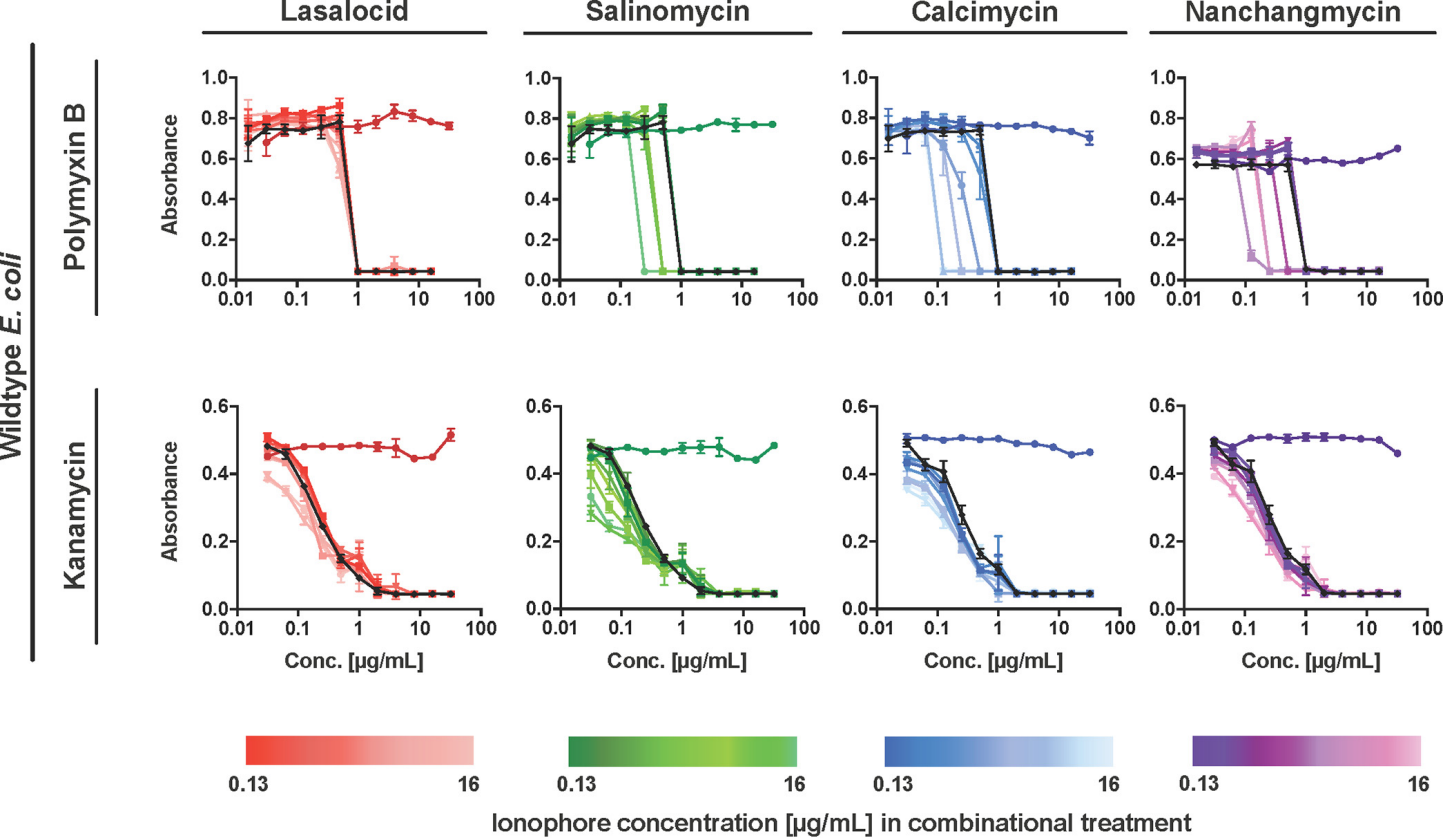
Combinatorial treatment in wild-type E. coli. E. coli were treated with either polymyxin B or kanamycin (black curves) in combination with selected ionophores, which were added as a fixed dosage (concentration range: 0.13 to 16 μg/mL). The antibiotics were tested as 2-fold serial dilutions up to 16 μg/mL (polymyxin B) or 32 μg/mL (kanamycin). The ionophores were tested as single treatments (lasalocid, dark red circles; salinomycin, dark green; calcimycin, dark blue; nanchangmycin, dark purple) as negative controls. Results are representative of two biological replicates, and data points are means ±SD (*n* = 3).

Polyether ionophores are generally regarded as being inactive against Gram-negative pathogens. Although their ability (documented in this study) to target drug-resistant, Gram-positive clinical isolates, as well as persister populations and bacterial biofilm, is undoubtedly appealing, their lack of activity against Gram-negative pathogens is a clear limitation of this compound class. It is likely that polyether ionophores cannot efficiently penetrate the outer membrane of Gram-negative bacteria and that this is the reason for their low activity. Although uptake efficiency, to the best of our knowledge, has not been experimentally determined for all of the polyether ionophores in Gram-negative strains, Guyot et al. (35) showed that calcimycin bound to Escherichia coli but never entered the cells. This lack of penetration is also supported by studies using E. coli mutant strains with increased outer membrane permeability that remain sensitive to polyether ionophores and by studies showing that the polymyxin derivative PMBN sensitizes E. coli to calcimycin (15, 36). We hypothesized that combinations with compounds such as polymyxin B, which specifically target the outer membrane and is known to increase permeability toward other antibiotics, might allow all of the ionophores to act against Gram-negative pathogens, including E. coli, and we therefore performed a systematic study of these effects. None of the ionophores had any stand-alone effect against E. coli (or Pseudomonas aeruginosa and Acinetobacter baumannii, Fig. S2), but salinomycin (fractional inhibitory concentration [FIC] 0.25), calcimycin (FIC 0.125), and nanchangmycin (FIC 0.25) all lowered the MIC value for polymyxin B in a dose-dependent manner (Fig. 7). Conducting the same experiment with an antibiotic with an intracellular target (kanamycin, ribosome) indicates that the outer membrane is in fact the only thing preventing ionophores from exerting their effect on Gram-negative bacteria. Interestingly, lasalocid did not have clear synergy with polymyxin B, an observation that we are not currently able to explain. Collectively, these data suggest that inclusion of polyether ionophores in antibiotic cocktails should be systematically tested in pre-clinical settings.

### Conclusion.

In this study, we have demonstrated that polyether ionophores, despite their joint, canonical characteristics as ion-transporters, are in fact quite heterogeneous with respect to their antibacterial properties. The compounds’ dependencies on cation concentrations are distinct, and some display the ability to eradicate bacterial biofilms and inhibit persister populations. We show that resistance to polyether ionophores is difficult to achieve and does not result in cross-resistance within the ionophore family. Very recently, Zhu et al. (37) reported equally promising persister and biofilm potency for the ionophore nigericin, a compound we had originally omitted due to its low solubility in our carrier dimethyl sulfoxide (DMSO). They demonstrated *in vivo* efficacy against S. aureus in a mouse model, emphasizing the potential of ionophores as antibiotics.

Finally, we show that combinations with polymyxin B can expand the antibiotic activity of the ionophores into also covering Gram-negative bacteria. This is particularly interesting given the increasing challenges associated with drug-resistant Gram-negative pathogens.

## MATERIALS AND METHODS

### Bacterial strains and culture conditions.

Staphylococcus aureus DSM 20231 was studied in most of the conducted experiments. Escherichia coli BW25113 (obtained from the Keio Collection, Coli Genetic Stock Center, Yale University), Pseudomonas aeruginosa DSM 19880, and Acinetobacter baumannii DSM 300007 were included in combinatory treatment experiments. S. aureus isolates from bloodstream infections were obtained from the Department of Clinical Microbiology at Aarhus University Hospital.

All strains were cultured in a defined minimal M9 (CM-M9) medium unless otherwise stated. This medium contains M9 broth (Sigma-Aldrich cat no. 63011, containing NH_4_Cl [18.7 mM], Na_2_HPO_4_ [42.3 mM], KH_2_PO_4_ [22 mM], and NaCl [8.6 mM]), glucose (1%), Casamino acids (1%), CaCl_2_·2H_2_O (0.1 mM), MgSO_4_·7H_2_O (2 mM), thiamine hydrochloride (1 mM), nicotinamide (0.05 mM), and trace metal solution TMS3 (1 mL/L) (pH 7.4).

### Polyether ionophores.

The chemical structures and selected properties of the polyether ionophores used in the current study can be found in Fig. S1. The compounds were obtained from the following sources: calcimycin, Alomone Labs (cat no. A-600); ionomycin, Alomone Labs (I-700); narasin, Sigma-Aldrich (N1271); salinomycin, Toku-E (S002); monensin, Toku-E (M083); maduramycin, Sigma-Aldrich (34069); and nanchangmycin, Sigma-Aldrich (SML2251). Lasalocid was prepared by isolating its sodium salt from the commercially available veterinary premix AVATEC, followed by acidic extraction with H_2_SO_4_ and crystallization from ethanol, but it can also be obtained from commercial suppliers (Sigma-Aldrich, cat no. 73996).

### MIC analysis.

MIC analysis was performed by a broth microdilution method in 96-well microtiter plates in accordance with CLSI recommendations. Briefly, a culture was grown aerated overnight (16 to 18 h, 140 rpm, 37°C) and adjusted to a final density of 5 × 10^5^ CFU/mL. Compound stocks were prepared in 2-fold serial dilutions in DMSO (final concentration < 5%). Bacteria were treated with compounds overnight (20 to 24h, 37°C), and growth was measured as absorbance at 600 nm using a Tecan NanoQuant Infinite M200 Pro Plate Reader. The absorbance value correlating to a difference visible with the unaided eye was determined to an absorbance of 0.07, and measurements above this value were deemed as growth when determining the MIC.

### Minimum bactericidal concentration analysis.

MBC analysis was performed after MIC analysis of all concentrations without detectable growth. Cells were pelleted in 96-well microtiter plates (15 min, 3,000 rpm), and pellets were resuspended in 0.9% NaCl. Next, 10 μL of the suspension was spotted onto LB agar and allowed to dissolve into the agar. A concentration with visible growth was included as positive control. Agar plates were incubated overnight (16 to 24 h, 37°C) and checked for growth. A bactericidal effect is represented by a 3-log_10_ reduction in density, corresponding to a 99.9% reduction. Thus, spots with less than 5 colonies indicate a bactericidal effect and were used to determine the MBC.

### Biofilm assay.

A culture was grown aerated overnight (16 to 18 h, 37°C, 140 rpm) and adjusted to 1 × 10^7^ CFU/mL. The adjusted culture was inoculated with a peg-lid (30 min, 37°C) to adhere biofilm cells onto pegs. The peg-lid was then transferred to fresh medium and allowed to grow overnight (24 h, 37°C) before treatment. Biofilm cells were treated for 24 h (37°C) in CM-M9 with compounds (dissolved in DMSO) in a 2-fold serial dilution prepared in medium-to-lower DMSO concentration. The peg-lid was washed twice in 0.9% NaCl and sonicated for 10 min in fresh medium, and biofilm cells were allowed to recover (72 h, 37°C). The MBIC was determined after 24 h of treatment, and the MBEC was determined after 72 h of recovery, both with absorbance measurement at 600 nm and visual inspection.

### Persister cell assay.

A culture was grown aerated overnight (22 to 25, h, 37°C, 140 rpm) in tryptic soy broth (TSB), diluted 1:1,000 in fresh TSB, and incubated overnight a second time. The culture was adjusted to an optical density at 600 nm (OD_600_) of 1.0 and washed using a modified CM-M9 medium (without glucose and Casamino acids). In a 96-well microtiter plate, compounds were dispensed in modified CM-M9 at 50× MIC, and the adjusted culture was added, yielding a final density of OD_600_ = 0.1. The plate was incubated overnight (20 to 24 h, 37°C), and pellets were washed by pelleting (10 min, 14,000 rpm) in a tabletop centrifuge in Eppendorf tubes to remove compounds. The cells were finally 10-fold serially diluted in modified CM-M9, and 10 μL from dilutions 10^0^ to 10^7^ was spotted onto LB agar. On the following day, colonies were counted for CFU/mL enumeration.

### Time-dependent killing.

For early exponential phase, a culture was grown aerated overnight (16 to 18 h, 37°C, 140 rpm) in CM-M9 medium, adjusted to 1 × 10^5^ CFU/mL and grown to the early exponential phase (3 h, 37°C, 140 rpm) before treatment. For late exponential phase, a culture was grown aerated overnight (16 to 18 h, 37°C, 140 rpm) in CM-M9 medium and used directly for treatment. In both cases, the cultures were diluted 1:10 with CM-M9 and treated with compounds in a 96-well format at 10× MIC (CM-M9). At six chosen time points (0, 2, 4, 8, 12, and 24 h), samples were collected for CFU/mL enumeration. Here, the cells were washed (15 min, 3,000 rpm) in 0.9% NaCl in the 96-well plates and 10-fold serially diluted in 0.9% NaCl, and 10 μL from each dilution (10^0^ to 10^6^) was spotted onto LB agar. After incubation (16 to 24 h, 37°C), CFU/mL were enumerated.

### Resistance development by single-step method.

Agar plates were prepared with 1× MBC of the desired compound by addition of compound (dissolved in DMSO) to 50 to 60°C LB agar. Meanwhile, a culture was grown aerated overnight (16 to 18 h, 37°C, 140 rpm) and concentrated 20× by centrifugation (20 min, 4,000 rpm), yielding a final density of ~10^9^ CFU/mL. Next, 100 μL of the dense culture was plated onto the agar plates containing compound, and plates were incubated for 24 to 72 h (37°C) and checked for growth each day.

### Resistance development by sequential passaging.

An overnight culture (16 to 18 h, 37°C, 140 rpm) with a density of 1 × 10^8^ CFU/mL was diluted 1:40 and treated with five different compound concentrations (0.25×, 0.5×, 1×, 2×, and 4× MIC) in a 96-well microtiter plate (one plate per technical triplicate). The plates were incubated overnight (22 to 24 h, 37°C) and checked for growth. The new MIC value was determined with visual inspection, and 5 μL from the well with highest concentration with detectable growth (0.5× the new MIC value) was passed on and treated with the five compound concentrations (0.25× to 4× MIC) relative to the new MIC, diluting the culture 1:40. This was repeated for 25 days or until resistance had developed. Every day, an untreated growth control and medium blank were included.

### DNA extraction, library preparation, and sequencing of *S. aureus* AUH1-8.

DNA was extracted using the DNeasy UltraClean Microbial kit (Qiagen) and quantified by a Qubit fluorometer (Thermo Fisher Scientific). Oxford Nanopore Technologies (ONT) libraries were prepared by multiplexing using the Rapid Barcoding Sequencing kit (SQK-RBK110.96, ONT). Libraries were loaded onto a FLO-MIN106 R9.4.1 flow cell and sequenced on a MinION instrument using MinKNOW 22.12.7 (ONT). Basecalling was performed with Guppy v.6.4.6.

### Genome reconstruction and typing of *S. aureus* AUH1-8.

Reads were demultiplexed and adaptors trimmed with porechop v.0.2.4. The trimmed reads were assembled into contigs with Flye v.2.9. The contigs were polished with medaka v.1.5.0 and the polished assemblies were annotated with Prokka v.1.14.5. The assemblies were typed and encoded antimicrobial resistance genes were identified using the CLC Microbial Genomics Module v.21.1.1 (Qiagen). Typing was done with the workflow “Type with large MLST scheme 1.2” with the parameters kmer size 21, minimum kmer ratio 0.2, typing threshold 0.99, search novel alleles, minimum required fraction of kmers 0.9, minimum length 50, and minimum length fraction 0.8. AMR genes were identified with the workflow “Find resistance with nucleotide DB 1.2” with the parameters minimum identity 98%, minimum length 60%, and filter overlaps. The genomic data have been submitted to GenBank under BioProject accession no. PRJNA961647.

### Isolation of genomic DNA and DNA sequencing.

The genomic DNA (gDNA) was isolated from the nanchangmycin-resistant strains to perform DNA sequencing. For isolation of gDNA, the Monarch Genomic DNA purification kit was used (T3010S) together with bead-beating with zirconia/silica beads. The procedure for the Monarch kit was followed with a few adjustments, as follows. Overnight cultures (16 to 18 h, 37°C, 140 rpm) of each resistant strain and wild-type S. aureus DSM 20231 were grown to densities of ~1 × 10^8^ CFU/mL and pelleted (1 min, 14,000 rpm). Pellets were resuspended in Tris-buffer and transferred to bead columns. Samples were bead-beaten (40 sec, 6.0 m/s), beads were pelleted (10 min, 12,300 × *g*), and supernatant was stored in the freezer overnight. The samples were then treated with enzymes: first lysozyme (20 mg/mL, 1 h, 37°C), then proteinase K (25 μL, 2 h, 56°C, 1,400 rpm) and finally RNase A (3 μL, 10 min, 56°C, 1,400 rpm). The lysed samples were then processed as described in the Monarch protocol for gDNA binding, washing, and elution. The samples were prepared for sequencing using the Nextera XT DNA Library Preparation kit (Illumina) and paired-end sequenced (2 × 300 bp) on a MiSeq sequencer using the MiSeq Reagent kit V3 (Illumina). Geneious was used to trim the sequencing data (using BBduk) and then mapped onto the reported genome of S. aureus DSM 20231 (NZ_CP011526). All data have been deposited in GenBank under BioProject accession no. PRJNA877072 and the following accession numbers: CP104478 (untreated reference), CP104477 (resistant mutant 1), CP104476 (resistant mutant 2), and CP104475 (resistant mutant 3).

### Design of experiment approach.

Using the design of experiment (DOE) approach, we studied the ionophores’ bioactivity in the presence of different cation concentrations. To test the desired concentrations, a modified version of the CM-M9 medium was prepared containing NH_4_Cl (1 g/L), Na_2_HPO_4_ (7.5 g/L), and NaH_2_PO_4_ (3 g/L) instead of the M9 broth. The desired concentrations of NaCl, KCl, CaCl_2_, and MgSO_4_ could then be added. We wanted to test 17 different cation combinations and thus prepared 17 different media.

A culture was grown aerated overnight (16 to 18 h, 37°C, 140 rpm) and diluted to a final density of 5 × 10^5^ CFU/mL. The bacteria were treated with three different compound concentrations (0.25×, 1×, and 4× MIC) in the 17 different types of media in 96-well microtiter plates. Plates were incubated overnight (20 to 24 h, 37°C), and growth was measured by absorbance at 600 nm. A plate with blank media was also prepared and these absorbance values were subtracted from the growth values, giving the final growth responses. These values were plotted into the MODDE pro program, which performs statistical analysis showing how each cation affects growth, among other features.

### Fluorescence with 3,3′-dipropylthiacarbocyanine iodide.

The ionophores’ effect on membrane potential was studied using the fluorogenic probe 3,3′-dipropyl-thiacarbocyanine iodide (DiSC_3_(5)).

A culture was grown aerated overnight (16 to 18 h, 37°C, 140 rpm), diluted to OD_600_ = 0.1, and incubated to early exponential phase (3 h, 37°C, 140 rpm). The culture was washed (4,000 rpm, 15 min, 4°C) three times in a sucrose buffer containing K_2_HPO_4_ ·3H_2_O (10 mM), MgSO_4_ ·7H_2_O (5 mM), and sucrose (250 mM) (pH 7.0). After the final wash, the pellet was resuspended in the buffer supplemented with 0.1 M KCl to a final density of OD_600_ = 0.3.

A culture plate was prepared in a black 96-well plate (Greiner Bio-One 96-well μClear, black with clear bottom) in which the diluted culture was loaded with 1% of fluorescent dye DiSC_3_(5), yielding a final concentration of 1 μM DiSC_3_(5). The dye was allowed to stabilize for 15 min in the dark before fluorescence was measured (excitation 620 ± 10 nm, emission 685 ± 10 nm, gain 2,600, focal height 7.3 mm, top optic, and linear shaking of 500 rpm, 10s). In another black 96-well plate, compounds were dispensed as a 20× solution. Compounds were tested at 10× MIC, so stock solutions of 200× MIC were prepared in DMSO (final concentration = 5%). After the dye was stabilized inside the bacterial membrane, the culture loaded with dye was transferred to the compound plate, yielding the final concentration of 10× MIC. Fluorescence was measured for a total of 60 min. Controls of DMSO (5%) and buffer were included.

### Combinatorial treatments.

For combinatorial treatments, the experiments were performed as described in the “MIC analysis” section, but with two compounds instead of one. The antibiotics were tested in 2-fold serial dilutions up to 16 μg/mL for polymyxin B and cloxacillin, or up to 32 μg/mL for kanamycin, and the ionophores were added at fixed dosages for each serial dilution. Eight different combinations were made, testing the ionophores in the concentration range of 0.125 to 16 μg/mL. The ionophores and antibiotics were also tested as single treatments as negative and positive controls, respectively.

### Screening of Nebraska Transposon Mutant Library.

The Nebraska Transposon Mutant Library is stored in glycerol in 96-well microtiter plates at −80°C. Material from the frozen stock was transferred with a Deutz 96 Cryo-replicator from the 96-well microtiter plates onto TSB agar plates supplemented with 0.5 μg/mL of either lasalocid or salinomycin, 0.0625 μg/mL calcimycin, or 0.125 μg/mL calcimycin, corresponding to 1/16 MIC of the MRSA JE2 wild-type strain in TSB. The plates were incubated at 37°C for 24 h and visually inspected for lack of growth of individual mutants. MICs of individual mutants were determined in TSB in a 96-well format and determined as the lowest concentration with no visible growth.
