# Supplementary material for: Polyether Ionophore Antibiotics Target Drug-Resistant Clinical Isolates, Persister Cells, and Biofilms
Source: Microbiol Spectr. 2023 Jun 8;11(4):e00625-23. doi: 10.1128/spectrum.00625-23 (PMC10433871; doi:10.1128/spectrum.00625-23)
Supplement: Supplemental file 1 — Fig. S1 and S2 and Tables S1 to S4. Download spectrum.00625-23-s0001.pdf, PDF file, 0.5 MB [file spectrum.00625-23-s0001.pdf]

Supporting Information

Polyether ionophore antibiotics target drug-resistant clinical isolates, persister cells and biofilms

Malene Wollesen[1], Kasper Mikkelsen[3], Marie Selch Tvilum[2], Martin Vestergaard[3], Mikala Wang[4], Rikke L. Meyer[5,6], Hanne Ingmer[3], Thomas B. Poulsen[1,*], Thomas Tørring[2,*]

[1] Department of Chemistry, Aarhus University, Langelandsgade 140, DK-8000, Aarhus C, Denmark.
[2] Department of Biological and Chemical Engineering, Gustav Wieds Vej 10, DK-8000, Aarhus C, Denmark.
[3]Department of Veterinary and Animal Sciences, University of Copenhagen, Denmark
[4]Department of Clinical Microbiology, Aarhus University Hospital, Palle Juul-Jensens Boulevard 99, DK-8200 Aarhus N, Denmark
[5] Interdisciplinary Nanoscience Center, Gustav Wieds Vej 14, DK-8000, Aarhus C, Denmark.
[6] Department of Biology, Ny Munkegade 114-116, DK-8000, Aarhus C, Denmark

[*] e-mail: thomast@bce.au.dk; thpou@chem.au.dk

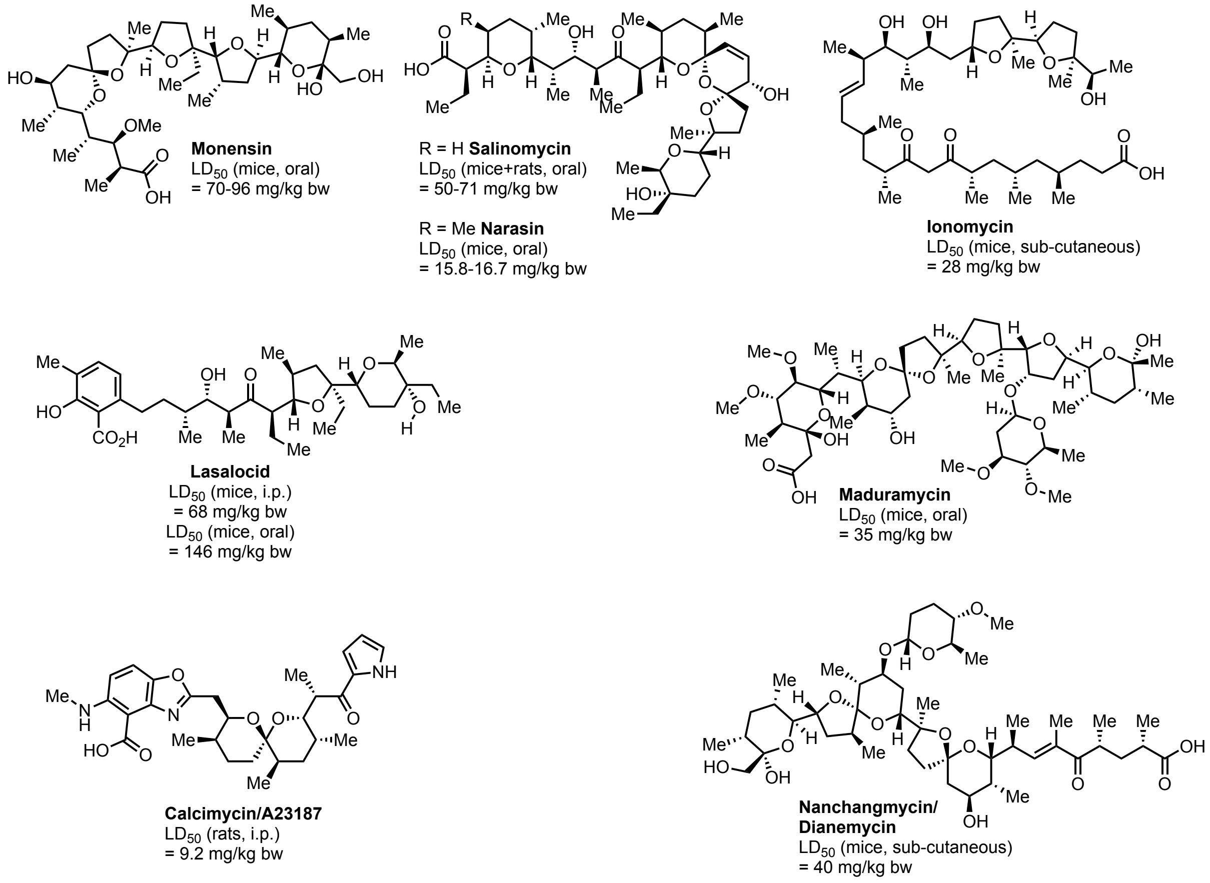

Fig. S1. Chemical structures of polyether ionophores used in this study and a compilation of selected, reported toxicity values (acute LD_{50}) in mice or rats. Additional data can be found in the parent resources: Monensin[1], salinomycin[2], narasin[3], Ionomycin[4], lasalocid[5], maduramycin[6], calcimycin[7], nanchangmycin[8].

[1] https://www.ema.europa.eu/en/documents/mrl-report/monensin-cattle-including-dairy-cows-summary-report-committee-veterinary-medicinal-products_en.pdf (accessed on 2/1-2023)
[2] https://www.efsa.europa.eu/en/efsajournal/pub/76 (accessed on 2/1-2023)
[3] https://www.efsa.europa.eu/en/efsajournal/pub/5460 (accessed on 2/1-2023)
[4] W.-C. Liu, *et al. J. Antibiot.* **1978**, *31*, 815-819.
[5] https://www.ema.europa.eu/en/documents/mrl-report/lasalocid-sodium-summary-report-committee-veterinary-medicinal-products_en.pdf (accessed on 2/1-2023)
[6] https://www.efsa.europa.eu/en/efsajournal/pub/1952 (accessed on 2/1-2023)
[7] T. J. Sobotka, R. F. Brodie, Y. Quander, M. O'Donnell, G. L. West, *Neurotoxicol. Teratol.* **1987**, *9*, 99-106
[8] R. L. Hamill, M. M. Hoehn, G. E. Pittenger, J. Chamberlin, M. Gorman, *J. Antibiot.* **1969**, *22*, 161-164.

Table S1. Biofilm eradication results in the clinical isolates. A biofilm in each strain was first grown for 24 hours and then treated for another 24 hours. The biofilms were allowed to recover for 72 hours, and the minimum biofilm eradication concentrations (MBEC) were determined and listed below in µg/mL. The assay was performed in five methicillin-sensitive strains and three MRSA strains. Clinical isolates 1, 2 and 5 are penicillin-resistant and methicillin-sensitive whereas clinical isolates 3 and 4 are both penicillin- and methicillin-sensitive. Clinical isolates 6-8 are methicillin-resistant. Wildtype *S. aureus* DSM 20231, and wildtype *S. aureus* MRSA USA 300 je2 were included for comparison.

	**Methicillin-sensitive *S. aureus***						**Methicillin-resistant *S. aureus***			
**Compound**	***S. aureus* DSM 20231**	***Clinical isolate 1***	***Clinical isolate 2***	***Clinical isolate 3***	***Clinical isolate 4***	***Clinical isolate 5***	***S. aureus* *MRSA* USA 300 je2**	***Clinical isolate 6***	***Clinical isolate 7***	***Clinical isolate 8***
Lasalocid	64-128	128	128	128	128	128	128	128	128	128
Salinomycin	≥1024	>1024	>1024	>1024	>1024	>1024	1024	1024	1024	1024
Monensin	>128	>128	>128	>128	>128	>128	>128	>128	>128	>128
Calcimycin	4-8	16	8	16	16	4	4	4	8	4
Ionomycin	≥1024	>1024	>1024	>1024	>1024	>1024	>1024	>1024	>1024	>1024
Narasin	256-512	1024	>1024	>1024	>1024	1024	256	256	256	256
Maduramycin	≥1024	>1024	>1024	>1024	>1024	>1024	>1024	>1024	>1024	>1024
Nanchangmycin	32	64	64	128	64	64	128	128	8	8
Vancomycin	1024	>1024	1024	>1024	>1024	>1024	>1024	>1024	>1024	>1024

Table S2. List of genes with mutations found in nanchangmycin-resistant isolates. The sequencing data have been deposited to Genbank under Bioproject number PRJNA877072 and accession numbers: CP104478 (untreated reference), CP104477 (resistant mutant 1), CP104476 (resistant mutant 2), CP104475 (resistant mutant 3).

**Locus**	**Name**	**Predicted function**
WP_000021864.1	TrkH	Potassium uptake
WP_001161085.1	MspA	membrane stabilizing
WP_000066900.1	SarV	Transcriptional regulator
WP_000757569.1	glycerophosphorodiesterase	Phosphodiester hydrolysis
WP_001151499.1	ATP-dependent helicase recG	DNA repair
WP_001118443.1	translation initiation factor IF-1	Protein synthesis

Table S3. Hits of the Nebraska Transposon Mutant Library screen, with corresponding MIC values of the relative gene-inactivated mutants against Lasalocid (Las), Salinomycin (Sal), Calcimycin (Cal), and Nanchangmycin (Nan).

Locus	Name	Product	Las	Sal	Cal	Nan
MRSA wildtype			4	2	0.0625	2
SAUSA300_1357	*aroC*	chorismate synthase	0.25	0.125	0.0625	0.25
SAUSA300_1615	*hemB*	delta-aminolevulinic acid dehydratase	0.5	0.25	0.0625	0.25
SAUSA300_0844	*ndh2*	NADH:ubiquinone reductase	2	1	0.0625	1
SAUSA300_0962	*qoxB*	quinol oxidase, subunit I	2	1	0.0625	1
SAUSA300_0961	*qoxC*	quinol oxidase, subunit III	2	1	0.0625	1
SAUSA300_0963	*qoxA*	quinol oxidase, subunit II	2	1	0.0625	1
SAUSA300_1016	*cyoE*	protoheme IX farnesyltransferase	2	1	0.0625	1

Table S4. Multi locus sequence typing of clinical isolates and identified resistance genes. MLST marked with * was not found in pubMLST so the closest match is listed. AUH3 and AUH4 appear to be identical even though they are isolated from different patients on difference dates.

Isolate ID	MLST	Resistance genes
AUH1	ST30*	BlaZ
AUH2	ST72	BlaZ
AUH3	ST5/ST4166	
AUH4	ST5/ST4166	
AUH5	ST1*	BlaZ
AUH6	ST6	MecA, BlaZ
AUH7	ST22/ST957	MecA, BlaZ
AUH8	ST8/ST4803	MecA, BlaZ

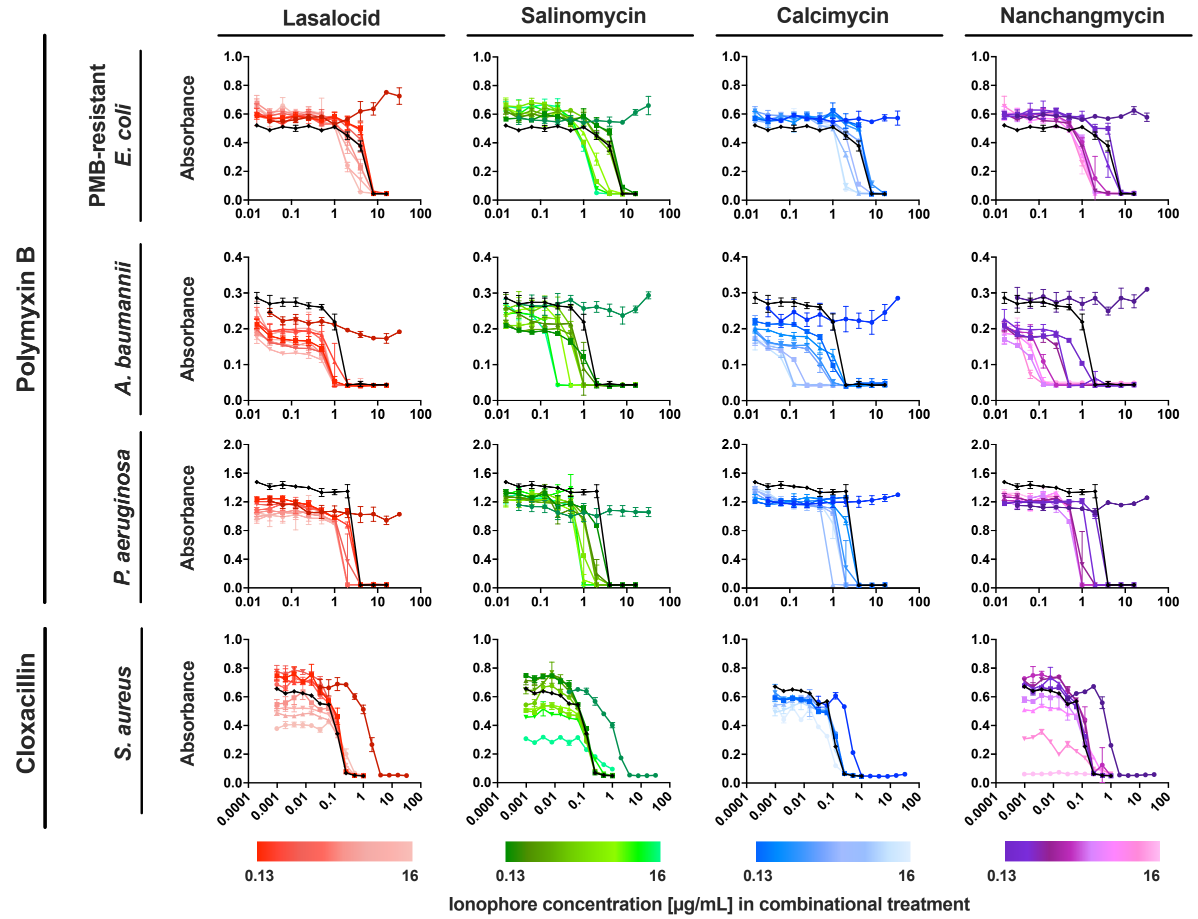

Fig. S2. Combinatorial treatment in a polymyxin-resistant *E. coli* (DH5α pGDP2 MCR-1), *A. baumannii* DSM 300007, *P. aeruginosa* DSM 19880, and *S. aureus* DSM 20231. The three gram-negative strains were treated with polymyxin B, and *S. aureus* was treated with cloxacillin, all in combination with four selected ionophores which were added as a fixed dosage (concentration range 0.13-16 µg/mL in gram-negative strains; 0.004-0.5xMIC in *S. aureus*). Polymyxin B, cloxacillin and the ionophores were tested as single treatments (PMB/cloxacillin: black; lasalocid: dark red circles; salinomycin: dark green circles; calcimycin: dark blue circles; nanchangmycin: dark purple circles) as positive and negative controls.
